# Use of 18F-FDG-PET/CT for Retroperitoneal/Intra-Abdominal Soft Tissue Sarcomas

**DOI:** 10.1155/2018/2601281

**Published:** 2018-07-02

**Authors:** Dao-ning Liu, Zhong-wu Li, Hai-yue Wang, Min Zhao, Wei Zhao, Chun-yi Hao

**Affiliations:** ^1^Sarcoma Center, Peking University Cancer Hospital & Institute, Beijing, China; ^2^Department of Pathology, Peking University Cancer Hospital & Institute, Beijing, China; ^3^Department of Nuclear Medicine, Peking University Cancer Hospital & Institute, Beijing, China

## Abstract

**Rationale:**

To assess the diagnostic value of 18F-FDG-PET/CT for different retroperitoneal soft tissue sarcomas (STS) and other similar tumors. To analyze the predictive value of 18F-FDG-PET/CT for histological grade and main prognostic factors.

**Methods:**

195 patients with 44 different diseases have been included. Relationship between SUVmax, Clinical, pathological, and prognostic information has been analyzed.

**Results:**

Malignant tumors do not show higher SUVmax than benign ones (*P*=0.443). We divided all 44 different diseases into two groups; SUVmax of group 1 is significantly higher than group 2 (*P* ≤ 0.001). The ROC curve suggests 4.35 is the cutoff value to distinguish groups 1 and 2 (sensitivity = 0.789; specificity = 0.736). SUVmax correlates with Ki-67 index, mitotic count, vascular resection, histological grade, and recurrent STS without considering pathological diagnosis (*P*=0.001, *P*=0.012, *P*=0.002, *P* ≤ 0.001, and  *P*=0.037, resp.).

**Conclusion:**

18F-FDG-PET/CT cannot simply distinguish malignant and benign tumors in retroperitoneal/intra-abdominal cavity; however, the SUVmax of malignant tumors, inflammatory pseudotumor, and PPGL group is higher than the SUVmax of benign tumors, lymph node metastasis, hematoma, and low malignant STS group. Guidance of “SUVmax location” may be helpful for biopsy and pathology dissection.

## 1. Introduction

Retroperitoneal and intra-abdominal sarcomas contain various soft tissue tumors and a wide prognostic range. Precise diagnosis of these sarcomas always plays a key role in treatment selection, especially in the application of compartment resection [1]. As STS are generally cured by adequate surgical resection, inaccurate diagnosis may cause unnecessary resection of innocent organs and extra risks. Judgment of malignancy is not accurate even using biopsy [2]. Moreover, some researchers resort to other preoperative examinations such as 18F-Fluoro-2-deoxy-D-glucose (18F-FDG) positron emission tomography (PET); however, the effort of 18F-FDG-PET/CT to distinguish extremity low-grade sarcomas and benign lesions is not fully paid off [3]. Unlike extremity STS, the special anatomical cavity contains many different pathological types that can mimic STS. There is yet no such comprehensive study regarding the use of 18F-FDG-PET/CT in retroperitoneal and intra-abdominal STS, considering the limitation of low incidence of STS [4]. As the establishment of the only sarcoma center in China, the abundant resource provides us an opportunity to afford such an analysis.

The use of 18F-FDG-PET/CT in oncology is based on the FDG accumulation in malignant tumor cells. 18F-FDG-PET/CT is initially used for diagnosis, staging, and therapy monitoring. The value for prediction of tumor biology and even prognosis has been found in recent researches [5, 6]. To evaluate the use of 18F-FDG-PET/CT in precise diagnosis and prognosis prediction, we try to correlate maximum standardized uptake value (SUVmax) with different pathologic diagnosis and prognostic factors. In recent researches, common STS prognostic factors are histological grade, tumor size, age, location, vascular resection, number of resected organs, Ki-67 index, and multifocality [7–9]. Among them, Ki-67 is a nuclear protein associated with cellular proliferation. Histological grade of the FNCLCC system has been widely used in prognostic prediction for most STS [10]. They will be perfect representative histological data for us to evaluate 18F-FDG-PET/CT.

## 2. Materials and Methods

### 2.1. Patients

195 patients with 44 different pathological diagnosis have been enrolled. All patients accepted surgical treatment and 18F-FDG-PET/CT in retroperitoneal and intra-abdominal soft tissue sarcoma center, Peking University Cancer Hospital, during a 4-year period (November, 2013, to December, 2017). All patients did not receive any antitumor treatment before the performance of 18F-FDG-PET/CT. Ethical approval and written informed consent have been obtained. Clinical, pathological, and prognostic information have been collected. Histological grade of STS cases without GIST has been reassessed by two experienced pathologists in accordance with the FNCLCC system [10]; the two pathologists were blinded to the findings of clinical and prognostic information.

These patients were divided into two different parts. The first part included 154 retroperitoneal/intra-abdominal STS patients, for whom the relationship among SUVmax, pathological diagnosis, tumor biology, and clinical characters would be analyzed. Then, 32 patients were excluded as per the inclusion and exclusion criteria listed below. The remaining 121 patients would be used to analyze the relationship between SUVmax and prognosis. The second part included 41 patients with benign tumors, psuedotumors, reproductive tumors, and other tumors. They are excellent cases for differentiation.

### 2.2. Inclusion and Exclusion Criteria

1. Patients whose preoperative diagnosis and postoperative pathology are soft tissue sarcoma will be included; others will not be included for survival analysis.
2. Patients do not receive any antitumor treatment before 18F-FDG-PET/CT examination.
3. All patients accept R0/R1 resection, and those who accept R2 resection will be excluded.
4. Expect for GIST, no distant metastasis is found before/during the operation.
5. All patients have signed the informed consent and agreed to participate in this study.
6. Those patients who died of perioperative complications or other noncancer-related causes will be excluded.

### 2.3. 18F-FDG-PET/CT Acquisition

Patients fasted for at least 6 h before the 18F-FDG-PET/CT scan. Images were acquired 1 h after injection of 3.7 MBq/kg 18F-FDG. Awhole-body scan (brain to midthigh) was performed with the patient in the supine position. CT exposure factors for all scans were 120 kV and 100 mAs. 18F-FDG-PET/CT images were reported in consensus by two experienced nuclear medicine physicians, who were blinded to the findings of clinical and prognostic information. At the same time, CT imaging was used to differentiate lesions from physiological uptake. The SUVmax of lesions were calculated. The SUVmax generated from each patient was used in the final analysis.

### 2.4. Statistics

Data collection and statistical analysis were performed with IBM SPSS Version 20 (SPSS Inc., Chicago, IL, USA). Enumeration data were expressed as mean and standard deviation, ranked data by cross-tabulation and percentages, and survival data by the Kaplan–Meier method. The ROC curve was used to find appropriate cutoff SUVmax for differentiation. For statistical analysis, *T* test, linear regression, ANOVA, nonparametric test, chi-square test, and log-rank test were employed. All tests were performed two-sided at a significance level of *P*=0.05.

## 3. Results

### 3.1. Diagnosis

**Figure 1:**
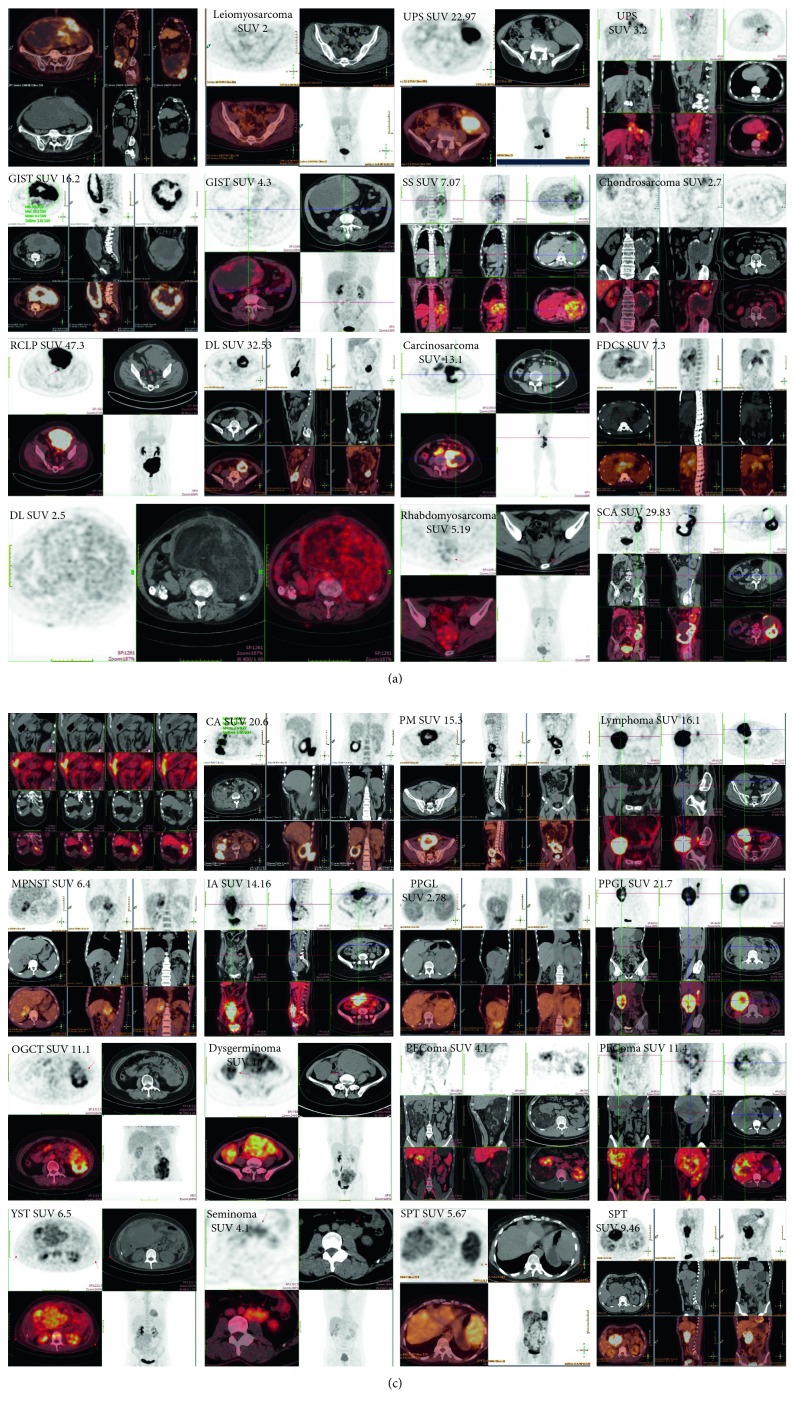
Representative cases in group 1. STS in group 1 are listed in (a). Other tumors in group 1 are listed in (b).

**Figure 2:**
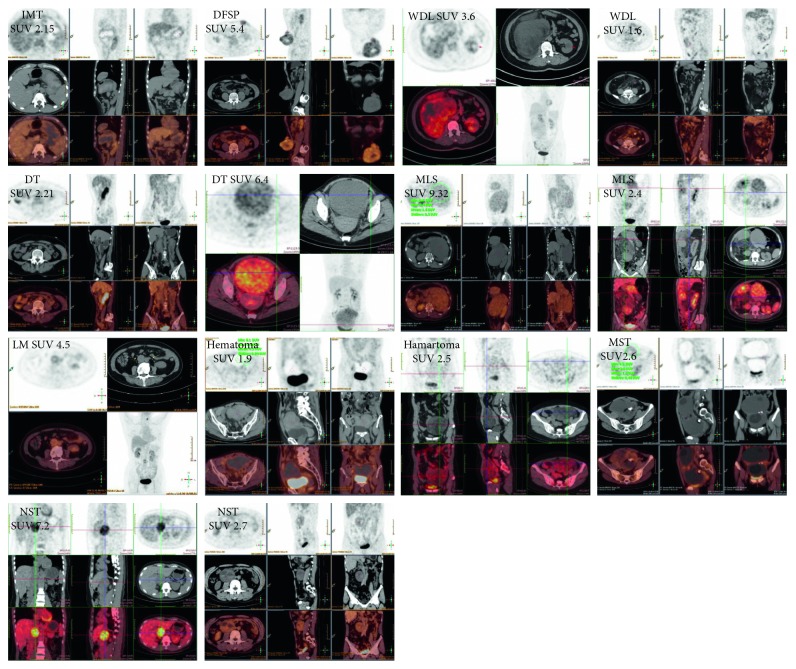
Representative cases in group 2.

**Figure 3:**
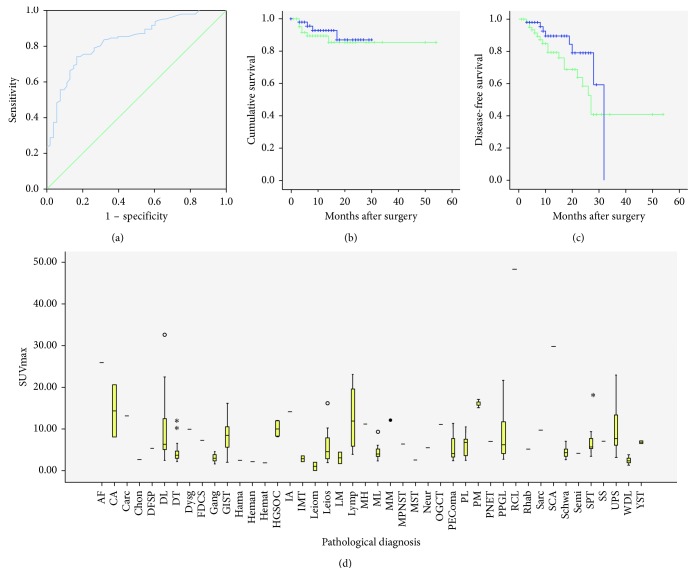
(a) The ROC curve for SUVmax to distinguish group 1 from group 2. (b, c) SUVmax (cutoff at 4.35) does not correlate with OS and DFS using Kaplan–Meier survival curves. (d) The box plot for SUVmax of all cases.

**Figure 4:**
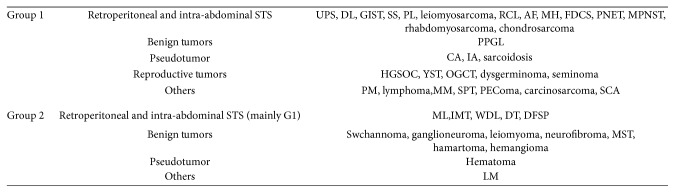
Specific pathological types for groups 1 and 2.

For all cases included, SUVmax correlates with Ki-67 index and mitotic count (*P*=0.001, and  *P*=0.012, resp.). Malignant tumors do not show higher SUVmax than benign ones (*P*=0.443). They have been divided into two groups according to the box plot, and literature review, representative images, and pathological types of each group have been shown in Figures 1–4. SUVmax of group 2 is significantly higher than group 1 (*P* ≤ 0.001). The ROC curve suggests 4.35 is an appropriate cutoff value to distinguish group 1 from group 2 (sensitivity = 0.789; specificity = 0.736, Figure 3). SUVmax of all diseases are listed in Table 1.

### 3.2. Treatment

For all STS cases, SUVmax correlates with vascular resection (*P*=0.002) but has no relationship with combined organs resection (cutoff at 3 organs, *P*=0.453). SUVmax does not correlate with pathological invasion of adjacent organs (*P*=0.085). SUVmax shows no relationship with operative time and blood loss (*P*=0.252  *and*  *P*=0.592, resp.).

### 3.3. Prognosis

Recurrent STS show higher SUVmax than primitive STS (*P*=0.037). SUVmax correlates with histological grade (*P* ≤ 0.001), grade 1 is the lowest and grade 3 is the highest. SUVmax for grade 1, 2 and 3 are 4.03 ± 2.28, 6.31 ± 4.78 and 10.09 ± 12.02, respectively. SUVmax also significantly correlates with tumor differentiation scores and tumor necrosis scores of the FNCLCC system (*P*=0.006  and  *P* ≤ 0.001, resp.). SUVmax for tumor differentiation scores 1, 2, and 3 are 3.51 ± 1.99, 5.47 ± 3.84, and 9.63 ± 7.89, respectively. SUVmax for tumor necrosis scores 0, 1, and 2 are 5.81 ± 3.94, 9.73 ± 8.57, and 11.28 ± 4.44. SUVmax shows no significant difference between multifocal and unifocal tumors (*P*=0.279). SUVmax does not correlate with tumor size (*P*=0.279). SUVmax shows no relationship with death or postoperative recurrence (*P*=0.081  and  *P*=0.162, resp.). Using 4.35 as the cutoff value, SUVmax does not correlate with DFS or OS by the Kaplan–Meier method (*P*=0.168  and  *P*=0.491, resp., Figure 3).

## 4. Discussion

Precise preoperative diagnosis of retroperitoneal and intra-abdominal sarcomas is always a vital problem, since different pathological diagnosis would lead to completely different treatment and prognosis. In former studies, the intermediate and high-grade malignant lesions have significantly higher FDG-uptake than the low-grade and benign lesions, but 18F-FDG-PET/CT offered inadequate discrimination between the latter two groups [3, 11]. Some researchers also tried to find a cutoff to differentiate malignant from benign tumors; the sensitivity and specificity of 18F-FDG-PET/CT for detecting malignant versus benign lesions were 79% and 77% using SUV ≥ 2.0 and 60% and 86% using SUV ≥ 3.0, respectively [4].

In our study, we included 44 different diseases for differentiation. Unlike STS elsewhere, we found that 18F-FDG-PET/CT cannot simply distinguish benign and malignant tumors in retroperitoneal and intra-abdominal cavity. To solve this problem, we divided them into 2 different groups. With this method, we found that sensitivity and specificity for distinguishing 2 different groups are 0.789 and 0.736 using SUVmax ≥ 4.35. Group 1 stands for malignant tumors, inflammatory pseudotumor, and pheochromocytoma and paraganglioma (PPGL). Group 2 stands for benign tumors, relatively low malignant STS, lymph node metastasis, and hematoma. The theory behind this system is that some STS with relatively low malignancy including desmoid tumor, myxoid liposarcoma, and well-differentiated liposarcoma often show lower SUVmax [12, 13]. STS in group 2 are all assessed as FNCLCC grade 1 sarcoma, except for 1 myxoid liposarcoma patient (G2) and 1 desmoid tumor patient (G2). The SUVmax of the special myxoid liposarcoma and desmoid tumor are 4.3 and 6.47, respectively. We also have a special pleomorphic liposarcoma case assessed as grade 1, and its SUVmax is 2.5. We do not get enough proof to conclude dermatofibrosarcoma protuberans (DFSPs) and inflammatory myofibroblastic tumor (IMT) as the members of group 2, because we only have a bit G1 cases. In Aisheng Dong's study, the SUVmax of IMT was 10.9 ± 5.5, with a high variability of SUVmax among tumors ranging from 3.3 to 20.8 [14]. DFSP can also present high SUVmax [15]. Most reports about hematoma and lymph node metastasis focus on detection of lesions but not on differentiation with other diseases, so they did not list data of SUVmax [16, 17]. SUVmax of hematoma and lymph node metastasis has been reported as 3.4 and 6.3, but we still need more evidence [18, 19]. We just temporarily regard them as group 2 members. On the other side, the range of PPGL SUVmax is from 2.5 to 62.3 [20]. Combined with our data, we list it as the only benign tumor in group 1.

In the future, we think that the members of different groups may vary with the accumulation of cases. If we can establish such a mature system, it could be very helpful for the clinical use of 18F-FDG-PET/CT in retroperitoneal and intra-abdominal sarcomas. There will be 2 possibilities of this system. One is 2 different groups with certain diseases. The other is G1 sarcoma in one group, and rest sarcomas in another one. For now, we prefer the combination of these 2 possibilities, as certain disease is more likely to be of certain grade. If we can make the system mature, this differentiation must be very helpful for preoperative diagnosis combined with other examinations. For example, with exclusion of fat-containing lesions using MRI, we should be very careful to perform compartment resection for group 2 diseases without liposarcoma.

For diagnostic aspect, SUVmax correlates with the Ki-67 index, mitotic count, and histological grade without considering different pathological types, which is the same as extremity STS [21]. This result suggests that 18F-FDG-PET/CT may be helpful for preoperative biopsy and pathology dissection. For retroperitoneal sarcomas, it is reliable for core biopsy to determine the presence of a sarcoma, but it is difficult to correctly identify sarcoma subtype and grade [2, 22]. Reason for this difficulty is the heterogeneity of sarcoma, which can be solved by multiple site sampling after resection. However, even sequential biopsies before resection cannot offer precise diagnosis for STS. The relationship of anatomic pathology and nuclear medicine mentioned in Manuel Scimeca's study has drawn our attention [23]. With the guidance of “SUVmax location,” it may be helpful for core biopsy and pathology dissection to find the most representative part of a tumor. It is also possible to build a map of histological grade and different cell types. If the hypothesis is proved, it will reduce the number of biopsy and increase the accuracy of diagnosis and grade. The chaotic circumstance for STS diagnosis means that STS diagnosis and grade may vary with different biopsies, different samplings, or different pathologists. Even pathology of primary tumor and recurrent tumor in one patient could be different. Some relations may exist between different STS, like one STS changes into another one after several recurrences. However, we must know that any further studies or hypothesis must be established on accurate diagnosis and histological grade. With development of imaging fusion, we are convinced that the fusion of 18F-FDG-PET/CT and ultrasound will greatly enhance the accuracy of core biopsy and pathology dissection. This is also the aim for our further study.

For therapeutic aspect, SUVmax correlates with vascular resection but not with combined organ resection. This is because of our aggressive operative decisions. As there is high risk of thrombosis or bleeding, vascular resection is relatively passive. However, we will perform compartment resection even though some organs are “not really infiltrated” by tumors. At the same time, SUVmax does not correlate with pathological invasion of adjacent organs (*P*=0.085), but the relationship is more significant than organ resection (*P*=0.453). To some extent, we think SUVmax may be helpful to predict tumor infiltration and operative risks.

In prognostic aspect, SUVmax does correlate with STS prognostic factors including histological grade and recurrent tumors. However, we do not find the relationship among SUVmax, OS, DFS, death, and postoperative recurrence. This is because our follow-up is relatively short, and the median survival of STS is 103 months for R0 resection [24]. Our median follow-up is 10 months overall, with a range of 1 through 54 months. As SUVmax correlates with STS prognostic factors, we are convinced that we can get a positive result with enough follow-up in the future. For instance, G3 and recurrent sarcomas have higher SUVmax than G1 and primary sarcomas. G3 and recurrent sarcomas always leads to bad prognosis.

## 5. Conclusion

From our observation of retroperitoneal/intra-abdominal tumors, we draw the conclusion that 18F-FDG-PET/CT cannot simply distinguish malignant and benign tumors. We find that the SUVmax of malignant tumors, inflammatory pseudotumor, and PPGL group is higher than the SUVmax of benign tumors, lymph node metastasis, hematoma, and low malignant STS group. Guidance of “SUVmax location” may be helpful for biopsy and pathology dissection.

## Figures and Tables

**Table 1:** SUVmax for all diseases.

Pathological diagnosis	*N*	SUVmax
*Retroperitoneal and intra-abdominal STS*		
Undifferentiated pleomorphic sarcoma (UPS)	9	10.78 ± 6.72
Dedifferentiated liposarcoma (DL)	32	8.93 ± 6.42
Gastrointestinal stromal tumor (GIST)	23	8.51 ± 3.73
Synovial sarcoma (SS)	2	7.09 ± 0.02
Pleomorphic liposarcoma (PL)	6	5.90 ± 3.20
Leiomyosarcoma	14	5.77 ± 3.98
Desmoid tumors (DT)	19	5.76 ± 5.54
Myxoid liposarcoma (ML)	7	4.70 ± 2.33
Inflammatory myofibroblastic tumor (IMT)	2	2.83 ± 0.95
Well-differentiated liposarcoma (WDL)	9	2.48 ± 0.88
Round cell liposarcoma (RCL)	1	47.3
Adult fibrosarcoma (AF)	1	25.94
Malignant hemangiopericytoma (MH)	1	11.21
Follicular dendritic cell sarcoma (FDCS)	1	7.3
Primitive neuroectodermal tumor (PNET)	1	7
Malignant peripheral nerve sheath tumor (MPNST)	1	6.4
Dermatofibrosarcoma protuberans (DFSP)	1	5.4
Rhabdomyosarcoma	1	5.19
Chondrosarcoma	1	2.7

*Benign tumors*		
Pheochromocytoma and paraganglioma (PPGL)	13	7.87 ± 5.26
Schwannoma	6	4.56 ± 1.59
Ganglioneuroma	3	3.13 ± 1.53
Leiomyoma	2	1.05 ± 1.48
Neurofibroma	1	5.5
Mature cystic teratoma (MST)	1	2.6
Hamartoma	1	2.5
Hemangioma	1	2.2

*Psuedotumor*		
Chronic abscess (CA)	2	14.4 ± 8.77
Infection of actinomyces (IA)	1	14.16
Sarcoidosis	1	9.75
Hematoma	1	1.9

*Reproductive tumors*		
High-grade serous ovarian carcinoma (HGSOC)	2	10.1 ± 2.68
Yolk sac tumor (YST)	2	6.8 ± 0.42
Ovarian granulosa cell tumor (OGCT)	1	11.1
Dysgerminoma	1	10
Seminoma	1	4.1

*Others*		
Peritoneal mesothelioma (PM)	3	16.06 ± 0.92
Lymphoma	4	12.75 ± 8.56
Malignant melanoma (MM)	2	12.05 ± 0.35
Solid pseudopapillary tumor (SPT)	7	7.58 ± 4.93
Perivascular epithelioid cell tumor (PEComa)	3	5.97 ± 4.78
Lymph node metastasis (LM)	2	3.10 ± 1.98
Carcinosarcoma	1	13.1
Sarcomatoid carcinoma (SCA)	1	29.83

## Data Availability

The data used to support the findings of this study are available from the corresponding author upon request.
